# Readthrough-induced misincorporated amino acid ratios guide mutant-specific therapeutic approaches for two CFTR nonsense mutations

**DOI:** 10.3389/fphar.2024.1389586

**Published:** 2024-04-25

**Authors:** Aiswarya Premchandar, Ruiji Ming, Abed Baiad, Dillon F. Da Fonte, Haijin Xu, Denis Faubert, Guido Veit, Gergely L. Lukacs

**Affiliations:** ^1^ Department of Physiology, McGill University, Montréal, QC, Canada; ^2^ IRCM Mass Spectrometry and Proteomics Platform, Institut de Recherches Cliniques de Montréal, Montréal, QC, Canada; ^3^ Department of Biochemistry, McGill University, Montréal, QC, Canada

**Keywords:** cystic fibrosis, CFTR, premature termination codons, nonsense-mediated mRNA decay, stop codon readthrough, affinity purification, tandem mass spectrometry, CFTR modulators

## Abstract

Cystic fibrosis (CF) is a monogenic disease caused by mutations in the CF transmembrane conductance regulator (*CFTR*) gene. Premature termination codons (PTCs) represent ∼9% of CF mutations that typically cause severe expression defects of the CFTR anion channel. Despite the prevalence of PTCs as the underlying cause of genetic diseases, understanding the therapeutic susceptibilities of their molecular defects, both at the transcript and protein levels remains partially elucidated. Given that the molecular pathologies depend on the PTC positions in CF, multiple pharmacological interventions are required to suppress the accelerated nonsense-mediated mRNA decay (NMD), to correct the CFTR conformational defect caused by misincorporated amino acids, and to enhance the inefficient stop codon readthrough. The G418-induced readthrough outcome was previously investigated only in reporter models that mimic the impact of the local sequence context on PTC mutations in CFTR. To identify the misincorporated amino acids and their ratios for PTCs in the context of full-length CFTR readthrough, we developed an affinity purification (AP)-tandem mass spectrometry (AP-MS/MS) pipeline. We confirmed the incorporation of Cys, Arg, and Trp residues at the UGA stop codons of G542X, R1162X, and S1196X in CFTR. Notably, we observed that the Cys and Arg incorporation was favored over that of Trp into these CFTR PTCs, suggesting that the transcript sequence beyond the proximity of PTCs and/or other factors can impact the amino acid incorporation and full-length CFTR functional expression. Additionally, establishing the misincorporated amino acid ratios in the readthrough CFTR PTCs aided in maximizing the functional rescue efficiency of PTCs by optimizing CFTR modulator combinations. Collectively, our findings contribute to the understanding of molecular defects underlying various CFTR nonsense mutations and provide a foundation to refine mutation-dependent therapeutic strategies for various CF-causing nonsense mutations.

## 1 Introduction

The cystic fibrosis transmembrane conductance regulator (CFTR), an anion-selective channel, is indispensable for salt and water transport across secretory and resorptive epithelia, as well as for preserving the airway surface liquid (ASL) homeostasis (Cutting, 2015). Cystic fibrosis (CF) is an autosomal recessive genetic disorder affecting >100,000 people worldwide (Guo et al., 2022). Over 2100 *CFTR* variants have been identified (Cystic Fibrosis Mutation Database: http://genet.sickkids.on.ca), of which approximately one-third have been confirmed as CF-causing since they result in functional expression defects of CFTR at the plasma membrane (PM) (Clinical and Functional Translation of CFTR: http://cftr2.org). The CF mutations are classified into seven distinct classes: Class I—protein synthesis defect, Class II—maturation defect, Class III–gating defect, Class IV—conductance defect, Class V—reduced quantity, Class VI—reduced PM stability, and Class VII—impaired *CFTR* mRNA production owing to large deletions in the *CFTR* gene (De Boeck and Amaral, 2016); however they are often associated with a combination of molecular and cellular phenotypes (Veit et al., 2016).

Two types of CFTR modulators are currently used in the clinics: i) folding correctors that improve CFTR folding and PM expression (Van Goor et al., 2011; Taylor-Cousar et al., 2017) and ii) potentiators, which increase the channel open probability (Po) (Van Goor et al., 2009; Eckford et al., 2012; Jih and Hwang, 2013). The FDA-approved Trikafta drug is a combination of the corrector VX-661 (tezacaftor), the gating potentiator VX-770 (ivacaftor), and the dual-acting corrector and potentiator VX-445 (elexacaftor) (Heijerman et al., 2019; Middleton et al., 2019; Veit et al., 2021). The approval of Trikafta has been expanded to 177 CFTR mutants based on their functional responsiveness to cAMP-dependent protein kinase (PKA)-induced CFTR channel activation in Fisher Rat Thyroid (FRT) epithelia, heterologously expressing the channel (Lopes-Pacheco et al., 2021). Although the available drugs can profoundly alleviate the clinical symptoms in ∼80–90% of the CF population, they inefficiently restore the activity of CFTR mutants harboring nonsense and some splicing mutations, which account for approximately 10% of all mutations in CF (Clinical and Functional Translation of CFTR: http://cftr2.org).

Typically, premature termination codons (PTCs) in the coding region result in reduced or complete loss of protein biogenesis or the production of a truncated protein (Frischmeyer and Dietz, 1999; Kellermayer, 2006; Mort et al., 2008). This can be attributed to the combination of i) the high translation fidelity of stop codon recognition by the release factor complex (Mohler and Ibba, 2017), ii) the accelerated elimination of transcripts containing PTCs >50 nucleotides upstream of the last exon-exon junction by the nonsense-mediated mRNA decay (NMD) pathway (Nagy and Maquat, 1998; Linde et al., 2007a; Celik et al., 2015; Lejeune, 2017; Dabrowski et al., 2018; Kim and Maquat, 2019) and iii) the largely impaired functional expression of truncated proteins (Keeling and Bedwell, 2011), as well as the small amount of constitutive or drug-induced translational stop codon readthrough (SCR) products (Mangkalaphiban et al., 2021).

Both various genetic therapies and small-molecule pharmacophores are pursued to alleviate the cellular phenotype of PTCs in various model systems (Taylor-Cousar et al., 2023). Significant progress has been made by using gene editing, engineered nonsense suppressor tRNA, and *CFTR* gene or mRNA delivery approaches (Marquez Loza et al., 2021; Ko et al., 2022; Albers et al., 2023; Graeber and Mall, 2023; Kulhankova et al., 2023; Walker et al., 2023). Pharmacological suppression of PTCs usually requires both the inhibition of the NMD pathway and stimulation of near-cognate (nc-tRNA) binding to and the misincorporation of amino acids (a. a.) into newly synthesized polypeptide chains at the PTC (de Poel et al., 2022; Ko et al., 2022). As most PTCs are also associated with severe loss of CFTR transcripts, significant efforts are invested into non-specific and gene-specific NMD suppression in CF (de Poel et al., 2022; Kim et al., 2022).

SCR can be facilitated typically by aminoglycosides (e.g., G418 and gentamicin) and their synthetic derivatives (e.g., ELX-02) to promote error-prone translation leading to the recovery of limited amounts of functional full-length proteins, as demonstrated for PTCs identified in diseases such as CF, β thalassemia, hemophilia A (Rowe et al., 2011; Borgatti et al., 2020; Martorell et al., 2020; Crawford et al., 2021). Notably, ELX-02 can partially rescue G542X-CFTR in CF intestinal organoids and primary human bronchial epithelial cells (Crawford et al., 2021; Venturini et al., 2021). The G418-induced SCR of the W1282X- and G542X-CFTR could be further enhanced by the cereblon E3 ligase modulators CC-90009, which induce proteasomal degradation of eRF3a and eRF3b (Lee et al., 2022).

While the SCR efficiency follows the rank order of UGA > UAG > UAA (Manuvakhova et al., 2000; Schueren and Thoms, 2016; Pranke et al., 2023), the suppressor tRNA selection, and consequently, the missense mutation introduced into CFTR are influenced by the stop codon, its sequence context, as well as the secondary structure and dynamics of the mRNA (Mao et al., 2014; Dabrowski et al., 2015; Keedy et al., 2018; Wangen and Green, 2020). To predict the CFTR modulator responsiveness, there is a need for the identification of missense a. a. incorporation ratios into full-length CFTR harboring PTCs, as our present knowledge relies on results obtained in chimeric reporters, encompassing only the immediate sequence vicinity of CFTR PTCs (Roy et al., 2016; Xue et al., 2017; Pranke et al., 2018; Chen et al., 2023).

However, the identification and quantification of missense a. a.s, as well as their incorporation ratios, into the full-length CFTR harboring PTCs is imperative to accurately predict the CFTR modulator responses and optimize therapeutic interventions. To this end, we implemented an affinity purification technique coupled with tandem mass spectrometry (AP-M/MS) to identify the a. a. incorporation ratios during SCR of three full-length CFTR PTC mutants. Furthermore, we evaluated the relative efficacy of various combinations of CFTR modulator treatments with readthrough induction and NMD inhibition in airway epithelia.

## 2 Materials and methods

### 2.1 Antibodies and reagents

Forskolin and CFTR_Inh_-172 were purchased from Tocris. The mouse monoclonal Anti-HA antibody was obtained from Covance (clone: 16B12, MMS-101R, dilution 1:1,000). VX-770, VX-809, VX-661, and VX-445 were purchased from MedChemExpress. Bisdemethoxycurcumin (bDMC) and apigenin were from Sigma-Aldrich. The small-molecule corrector compounds 4172 and 3151 were acquired from Life Chemicals. The CFTR corrector C4 and the SMG1 inhibitor were provided by the Cystic Fibrosis Foundation. All other chemicals were procured from Sigma-Aldrich.

### 2.2 Cell culture and stable cell line generation

CFBE41o- cells, provided by D. Gruenert (University of California in San Francisco, USA), were maintained in MEM (Invitrogen) supplemented with 10% FBS (Invitrogen), 2 mM L-glutamine and 10 mM HEPES on human fibronectin-coated plastic dishes (Ehrhardt et al., 2006). CFBE41o- cell lines expressing inducible CFTR variants with a 3HA tag in the fourth extracellular loop were generated using the ClonTech pLVX-Tight-Puro lentivirus technology as previously described (Veit et al., 2012). For experiments, CFBE41o- cells were seeded at a density of 2 × 10^4^ cells/well into 96-well plates or 1 × 10^5^ cells/filter on 1.12 cm^2^ Snapwell filter supports (Corning). CFTR expression was induced with 50–250 ng/mL of doxycycline for at least 4 days. BHK-21 cells were cultured in DMEM/F12 (50:50) medium (Wisent) supplemented with 5% FBS. The cells were grown to at least 70% confluence before treatment with drugs. 16HBE14o-cells genome-edited to produce the homozygous CFF-16HBEge R1162X and W1282X-CFTR cell lines were obtained from the Cystic Fibrosis Foundation (Valley et al., 2019).

### 2.3 CFTR mRNA quantification

RNA isolation and quantification by RT-PCR analysis were done as described previously (Veit et al., 2012). CFTR variants, except W1282X, were detected using the following primer pair: forward AGT​GGA​GGA​AAG​CCT​TTG​GAG​T, reverse ACA​GAT​CTG​AGC​CCA​ACC​TCA. W1282X was detected using the following primers: forward AGC​ATT​TGC​TGA​TTG​CAC​AGT and reverse TGG​ATG​GAA​TCG​TAC​TGC​CG. In CFBE41o- cells, *GAPDH* was used to normalize between samples: forward CAT​GAG​AAG​TAT​GAC​AAC​AGC​CT and reverse AGT​CCT​TCC​ACG​ATA​CCA​AAG​T. In BHK-21 cells, *RPLP1* was used to normalize between samples: forward ACG​GAG​GAT​AAG​ATC​AAT​GCC and reverse CAG​ATG​AGG​CTC​CCA​ATG​TT.

### 2.4 PM density measurement

The PM density of 3HA-tagged CFTR variants was determined as described previously (Veit et al., 2012). Briefly, the CFTR surface expression was assessed by cell surface enzyme-linked immunosorbent assay (ELISA). PM density measurements were normalized with Alamar Blue cell viability assay (Invitrogen) or bicinchoninic acid (BCA) total protein concentration assay.

### 2.5 Readthrough assay

To directly measure the full-length readthrough CFTR following PTC suppression, we developed a streptavidin (SA)-coated 96 well black-plate based ELISA assay (NUNC 436016). BHK-21 or CFBE41o- cells were treated with various correctors and/or readthrough drugs for 16–24 h. The cells were then lysed in a buffer containing 20 mM Tris-HCl pH 8.0, 300 mM NaCl, 2 M Urea, 0.04% Triton X-100, 10 mM Iodoacetamide, and protease inhibitor cocktail (cOmplete EDTA-free Protease Inhibitor Cocktail tablets, Roche). The SA plates were pre-washed with 1× PBS buffer with 0.1% NP-40 and blocked with 1% BSA-PBS + 0.1% NP-40 for 15–30 min on ice. The lysates from wild-type (WT) and other mutants with various treatments were added to the wells and incubated for at least 2 h on ice with gentle shaking in a cold room. The plate was washed once with 1× PBS +0.1% NP-40. Denaturing buffer (6 M Urea, 0.1% NP-40/PBS) was added to the bound protein and incubated at RT for 5 min. The plate was then washed four times with 0.1% NP-40/PBS and incubated with 1% BSA-PBS +0.1% NP-40 for 20 min at RT. The primary antibody (anti-HA, 1:1000) was then added. The plate was incubated for 1 h at RT with gentle shaking. The plate was again washed six times with 0.1% NP-40/PBS, and the secondary antibody (anti-mouse Fab-HRP, 1:1000) was added. After 1 h incubation at RT on the shaker, the plate was washed six times with 0.1% NP-40/PBS and probed with Amplex UltraRed reagent (A36006, Invitrogen) using a fluorescence spectrophotometer (TECAN). The results were normalized against the total protein concentration determined by the BCA assay, and the amount of readthrough full-length protein was expressed as a percentage of the WT.

### 2.6 Short-circuit current measurements

Short-circuit current (I_sc_) measurement of polarized CFBE41o- and 16HBE14o- has been described previously (Veit et al., 2018). Briefly, the Snapwell filter-grown cells were mounted in Ussing chambers (Physiologic Instruments). The basolateral side was filled with Krebs-bicarbonate Ringer (KBR) buffer (140 mM Na^+^, 120 mM Cl^−^, 5.2 mM K^+^, 25 mM HCO_3_^−^, 2.4 mM HPO_4_, 0.4 mM H2PO_4_, 1.2 mM Ca^2+^, 1.2 mM Mg^2+^, 5 mM glucose, pH 7.4), which was mixed by bubbling with carbogen (95% O_2_ and 5% CO_2_). The currents were measured in the presence of a basolateral-to-apical chloride gradient generated by replacing NaCl with Na^+^ gluconate in the apical buffer. For CFBE41o-, the basolateral membrane was permeabilized with 100 μM amphotericin B (Sigma-Aldrich). The transepithelial voltage was clamped at 0 mV after compensating for voltage offsets. The current measurements were recorded at 37°C in the presence of 100 μM amiloride with the Acquire and Analyze package (Physiologic Instruments).

### 2.7 Affinity purification of HBH-tagged CFTR constructs

The HBH-CFTR-3HA variants were expressed and affinity-purified as described previously (Schnur et al., 2019). Briefly, CFBE41o- monolayers were grown on fibronectin-coated 10-cm dishes and induced with doxycycline (250 ng/mL) for 4 days post-confluency. BHK-21 cells were grown to 70% confluence and treated with drugs for 16–18 h before lysis. After treatments (3 µM VX-809, 5 μM C4, 5 µM 4172, 200 μg/mL G418, 16–24 h at 37°C), cells were washed and lysed with lysis buffer (2 M Urea, 0.4% Triton X-100, 300 mM NaCl; 20 mM Tris pH 8.0, 1 mM DTT) supplemented with protease inhibitors. The lysate supernatant was bound to Dynabeads^®^ MyOne™ Streptavidin C1 (Thermo Fischer Scientific), followed by a series of extensive washes. The bead-bound protein samples were placed in 50 mM ammonium bicarbonate supplemented with 0.01% DMNG (Anatrace) until digestion.

### 2.8 Sample preparation and tandem MS

The on-bead proteins were first diluted in 4 M Urea/50 mM ammonium bicarbonate/10 mM CaCl_2_.2H_2_O and 0.1% Protease MAX Surfactant (Promega). Urea concentration was reduced below 1 M and ProteaseMAX Surfactant at 0.02% for the on-bead chymotrypsin digestion performed overnight at 25°C. The samples were then reduced with 13 mM dithiothreitol at 37°C and, after cooling for 10 min, alkylated with 23 mM iodoacetamide at room temperature for 20 min in the dark. The supernatants were acidified with trifluoroacetic acid, and residual detergents and reagents were removed using MCX cartridges (Waters Oasis MCX 96-well Elution Plate) following the manufacturer’s instructions. After elution in 10% ammonium hydroxide/90% methanol (v/v), samples were dried with a Speed-vac, reconstituted under agitation for 15 min in 12 µL of 2% ACN-1% FA and loaded into a 75 μm i. d. × 150 mm, Self-Pack C18 column, installed on the Easy-nLC II system (Proxeon Biosystems). Peptides were eluted with a two-slope gradient at a flow rate of 250 nL/min. Solvent B was first increased from 1% to 31% in 115 min and then from 31% to 92% B in 16 min. The HPLC system was coupled to an Orbitrap Fusion mass spectrometer (Thermo Scientific) through a Nanospray Flex Ion Source. Nanospray and S-lens voltages were set to 1.3–1.8 kV and 50 V, respectively. Capillary temperature was set to 250°C. Full scan MS survey spectra (m/z 300–1400) in profile mode were acquired in the Orbitrap with a resolution of 120,000 with a target value at 3e5. A top 25 shotgun MS/MS analysis was performed using a non-exclusive targeted mass list containing 52 peptide ions of interest, including all conceivable amino acid combinations at the respective PTC sites of the three CFTR mutants. Parent ions with charge states above five and unassigned charge states were excluded for MS/MS fragmentation. The target ions selected for MS/MS analysis were fragmented in the HCD collision cell and analyzed in the linear ion trap with a target value at 2e4 and normalized collision energy at 30 V. The dynamic exclusion duration was set to 25 s after 2 M/MS events.

### 2.9 Peptide identification and quantification

The peak list was generated with Proteome Discoverer (version 2.1) using the following parameters: mass range: 500–6000 Da, no MS/MS spectral grouping, precursor charge: auto, and the minimum number of fragment ions: 5. The data were searched against the UniProt human and user-defined CFTR mutant database using Mascot 2.6 (Matrix Science), with the mass tolerances for precursor and fragment ions set at 10 ppm and 0.6 Da, respectively. Other filters included one missed tryptic cleavage, fixed modifications of cysteine carbamidomethylation, and variable modification of methionine oxidation.

Manual validation of peptides was performed using Scaffold (version 4.8 or version 5). In alignment with existing literature, the amino acids R, C, and W were identified as the misincorporated amino acids at the PTC site. The quantification of UGA codon-associated peptide variants was performed by determining the relative abundance of each amino acid incorporation—R, C, and W. In particular, the quantification involved expressing the spectral counts of peptides with a specific amino acid variant as a fraction of the total spectral counts for peptides containing R, C, and W, using the formula:

$$\text{Relative abundance of }a\text{ specific variant}=\frac{\text{Spectral}\;\text{counts}\;\text{of}\;\text{peptides}\;\text{containing}\;a\;\text{particular}\;\text{variant}}{\text{Total}\;\text{spectral}\;\text{counts}\;\text{of}\;\text{peptides}\;\text{containing}\;R,C,\text{and}\;W}$$

### 2.10 Determination of tRNA abundance of BHK-21 cells

BHK-21 cells were maintained in DMEM/F-12 (5% fetal bovine serum [FBS]) (Wisent) at 37°C and 5% CO_2_. Cells were grown to 70% confluency, and total RNA was isolated using the TRIzol method according to the manufacturer’s protocol (Qiagen) with some modifications as described previously (Polte et al., 2019). RNA integrity was assessed via total RNA Analysis at nanogram sensitivity with the Agilent 2100 Bioanalyzer. To fully deacylate tRNAs, 5 μg of total RNA was incubated for 45 min at 37 °C in 100 mM Tris-HCl buffer (pH 9.0). Deacylated samples were purified by precipitation with ethanol and one volume of 100 mM NaOAc (pH 4.8), supplemented with 100 mM NaCl and glycogen (20 mg/mL). For subsequent normalization of arrays, each sample was spiked with three or four *in-vitro* transcribed tRNAs (2 μM of each), which do not cross-hybridize with human tRNA. Fluorescently labeled RNA:DNA hairpin oligonucleotides were ligated to deacylated tRNA samples using T4 DNA ligase (NEB) for 1 h at room temperature. For comparison, HEK-293 cells were used and labeled with Atto647 oligonucleotides, whereas other samples were typically labeled with Cy3-labeled oligonucleotides. Labeled tRNAs were extracted using phenol/chloroform/isoamyl alcohol (Roth) and precipitated with ethanol. The efficiency of ligation was analyzed using 10% denaturing PAGE, and a comparison of fluorescent signals to total tRNA was visualized by staining with SYBR gold (Invitrogen). Approximately 1–2 μg of labeled tRNAs from analyzed samples and HEK-293 were simultaneously hybridized for 16 h at 60 °C on a microarray chip containing 24 technical replicates of each full-length tDNA as previously described (Kirchner et al., 2017). Absolute tRNA concentration in HEK-293 cells was used as a baseline set to convert tRNA isoacceptor abundancies from comparative microarrays into absolute units represented as a fraction of total tRNA (Polte et al., 2019). For tRNA isoacceptors pairing to more than one codon, the fraction per codon was determined using the corresponding codon usage index. Values for codons read by more than one tRNA were summed. The fractions of all tRNAs for one species were normalized to 100%. The data are the mean of three biological replicates.

### 2.11 Statistical analysis

All statistical analyses were performed using GraphPad Prism 6.0. Data are expressed as mean ± s. e.m. from at least three independent experiments unless otherwise specified. Statistical significance for PM density measurements was evaluated using one-way ANOVA or unpaired t-tests unless mentioned otherwise. *p* < 0.05 was considered statistically significant. Statistical significance is indicated as follows: *, *p* < 0.05; **, *p* < 0.01; ***, *p* < 0.001.

## 3 Results

### 3.1 Isolation of full-length CFTR PTC variants generated by G418-induced stop codon readthrough

G542X, R1162X, S1196X, and W1282X, with allelic frequencies of 2.54%, 0.46%, 0.01%, and 1.22%, respectively (Clinical and Functional Translation of CFTR: http://cftr2.org), are among the most frequent PTCs in the *CFTR gene*. Readthrough therapy aiming for full-length protein production is a potential treatment option for these mutants. However, near-cognate tRNA incorporation that leads to missense mutations may introduce folding and/or gating defects, depending on the structural context of the mutated residue. Although several publications have explored the a. a. incorporation in chimeric systems of varying lengths, the identification and quantification of these misincorporated residues has not been attempted in the context of full-length CFTR (Roy et al., 2015; Roy et al., 2016; Pranke et al., 2017; Xue et al., 2017; Yeh and Hwang, 2020). Nevertheless, the sequence context could influence both the identity and frequency of the a. a. Insertion during PTC suppression (Wangen and Green, 2020).

To determine the a. a. incorporation into the full-length channel, we expressed G542X, R1162X, S1196X, and W1282X-CFTR tagged with the triple HA-tag (3HA) in the fourth extracellular loop (Sharma et al., 2004) and a COOH-terminal hexahistidine-biotin-hexahistidine (HBH)-tag (Okiyoneda et al., 2018; Schnur et al., 2019). The latter epitope tag allowed the selective isolation of full-length proteins using streptavidin affinity pulldown upon nonsense suppression to obtain sufficient amounts for identification of the a. a. incorporation at the PTC sites by tandem MS. To avoid NMD-mediated degradation, we expressed the mutant CFTR cDNA constructs without exon-junctions in baby hamster kidney (BHK-21) cells and used correctors targeting distinct CFTR domains to promote processing (Veit et al., 2018).

**FIGURE 1:**
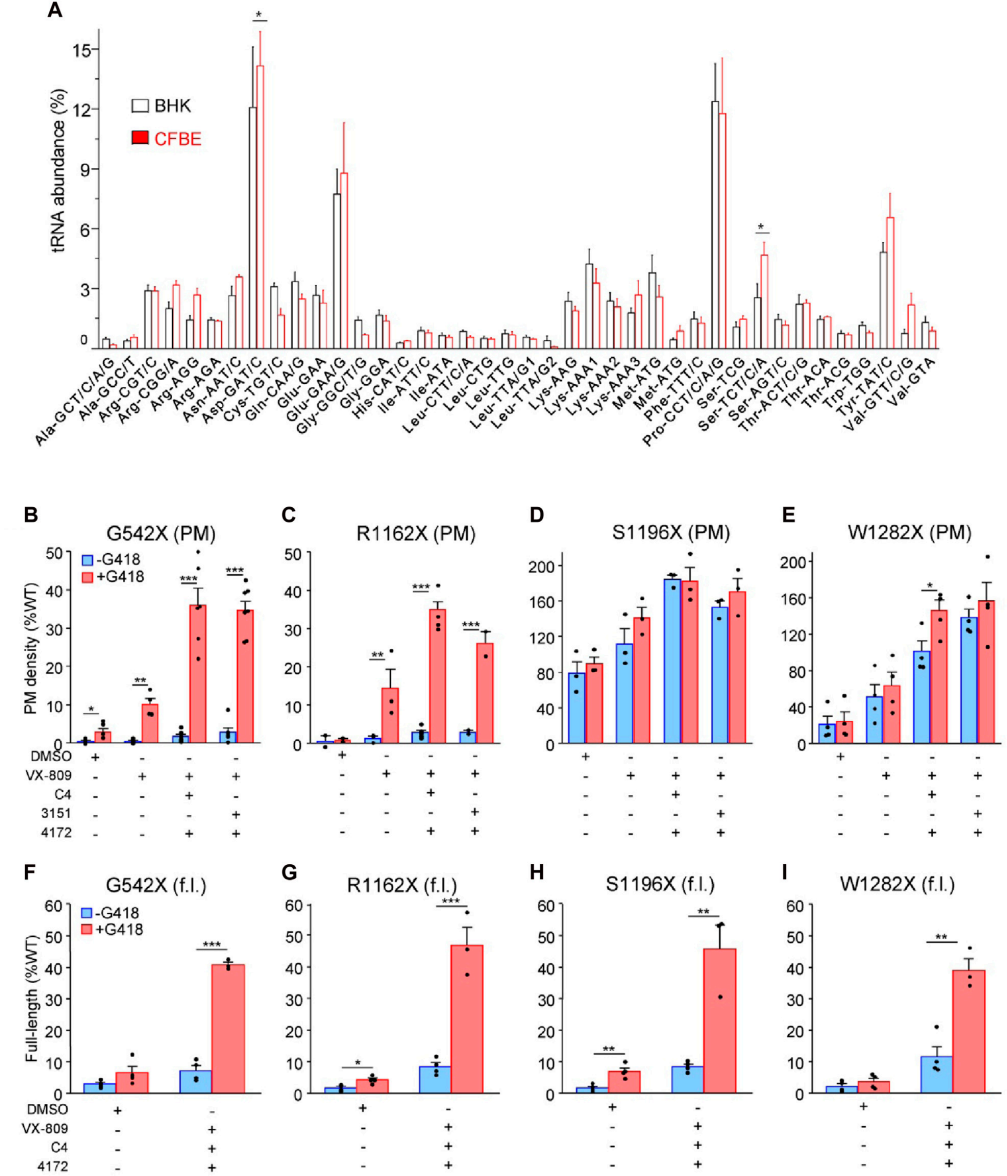
PTC suppression and rescue of resulting missense variants in mammalian cells. **(A)** Microarray analysis of absolute tRNA concentration in BHK-21 and CFBE41o- cells. Values from comparative tRNA microarrays were converted into absolute concentrations using comparative arrays *versus* HEK-293 cells and are represented for each codon as a fraction of total tRNA. tRNA isoacceptors are depicted with their cognate codon and the corresponding amino acid. The tRNAomes of HEK-293 and CFBE41o^−^ cells have been published (Kirchner et al., 2017; Polte et al., 2019). Data are presented as means ± s. e.m. (n = 3). **(B–E)** Plasma membrane (PM) expression of CFTR truncations before (blue) and after (red) PTC suppression upon treatment with mono- and triple correctors in BHK-21 normalized to *CFTR* mRNA expression. Cells were treated with correctors (3 µM VX-809, 5 μM C4, 10 µM 3151, 5 µM 4172, 16–18 h at 37°C) and/or G418 (200 μg/mL) or combinations thereof (n = 2–7) followed by PM density determination as described in Material and Methods. **(F–I)** The extent of readthrough measured as the amount of full-length (f.l.) protein bound to streptavidin-coated 96-well plates before (blue) and after (red) PTC suppression upon treatment with triple correctors in BHK-21 normalized to *CFTR* mRNA expression. Cells were treated with correctors (3 µM VX-809, 5 μM C4, 5 µM 4172, 16–18 h at 37°C) and/or G418 (200 μg/mL) or combinations thereof. BHK-21 cells were lysed with “Lysis buffer,” and 100 µL volume of the lysate from each cell line was incubated on streptavidin plates for 1–2 h, followed by detection of the 3HA-tag (n = 3–4). The data in b-i are presented as means ± s. e.m. of the indicated number of independent experiments. *, *p* ≤ 0.05; **, *p* ≤ 0.01; ***, *p* ≤ 0.001 by unpaired, two-tailed Student’s t-test.

While BHK-21 cells have been widely used for CFTR processing studies (Bagdany et al., 2017; Soya et al., 2023), this cell model may introduce a bias if its tRNA abundance differs from that of the human bronchial epithelial (CFBE41o-) cells. Using tRNA microarrays (Kirchner et al., 2017), we compared the tRNA abundance between BHK-21 and the human CFBE41o- cells. The relative abundance of the a. a. tRNAs, encoding for arginine (Arg), cysteine (Cys), and tryptophan (Trp), which are candidate residues incorporating in place of the UGA during the SCR (Roy et al., 2016; Xue et al., 2017; Pranke et al., 2018), did not significantly differ between the 2 cell lines (Figure 1A), validating the use of BHK-21 cells in place of human bronchial epithelial cells for our MS experiments.

Exposure to VX-809 alone and in combination with preclinical type-II and -III folding correctors with G418-induction of SCR resulted in PM expression of G542X-CFTR and R1162X-CFTR corresponding to approximately 35% of the WT (Figures 1B, C). The PM density of CFTR mutants was measured by PM ELISA. The corrector combination amplified the VX-809 effect by two to three fold (Figures 1B, C). The surface expression of S1196X and W1282X, however, could be restored to >100% of the WT (Figures 1D, E). The improved biochemical rescue susceptibility of these PTCs, which completely (S1196X) or partly (W1282X) lack the nucleotide-binding domain 2, is explained by their largely preserved folding capacity in the ER (Cui et al., 2007; Du and Lukacs, 2009; Haggie et al., 2017) and does not depend on G418-mediated SCR (Figures 1D, E). Notably, as the 3HA epitope tag is N-terminal of the R1162X, S1196X, and W1282X truncations, our PM density assay *per se* cannot differentiate between the increased expression of truncated and the full-length readthrough CFTR.

To distinguish between truncated and readthrough PTC CFTR variants, we implemented a streptavidin affinity isolation technique, utilizing the C-terminally attached HBH-tag for the detection of full-length CFTR variants. The CFTR-HBH was immobilized on 96-well streptavidin plates and quantified using an ELISA with anti-CFTR antibodies (Okiyoneda et al., 2018). This assay revealed a yield of full-length G542X, R1162X, and S1196X-CFTR corresponding to ∼35–45% of the WT-CFTR normalized for their mRNA levels (Figures 1F–H). Despite a similar full-length yield for W1282X, due to the lower mRNA expression level (Supplementary Figure S2A), the amount of isolated protein was insufficient for subsequent MS/MS analysis. These results suggest that the increased PM expression of the G542X and R1162X mutants is predominantly a consequence of the G418-mediated nonsense suppression. In contrast, the majority of the PM detected S1196X- and W1282X-CFTR constitute truncated molecules that escaped the ER-associated quality control degradation, and that can be increased by 2–5-fold upon folding corrector combination treatment as shown before (Du and Lukacs, 2009; Haggie et al., 2017).

### 3.2 Identification of full-length missense CFTR variants resulting from PTC suppression

**FIGURE 2:**
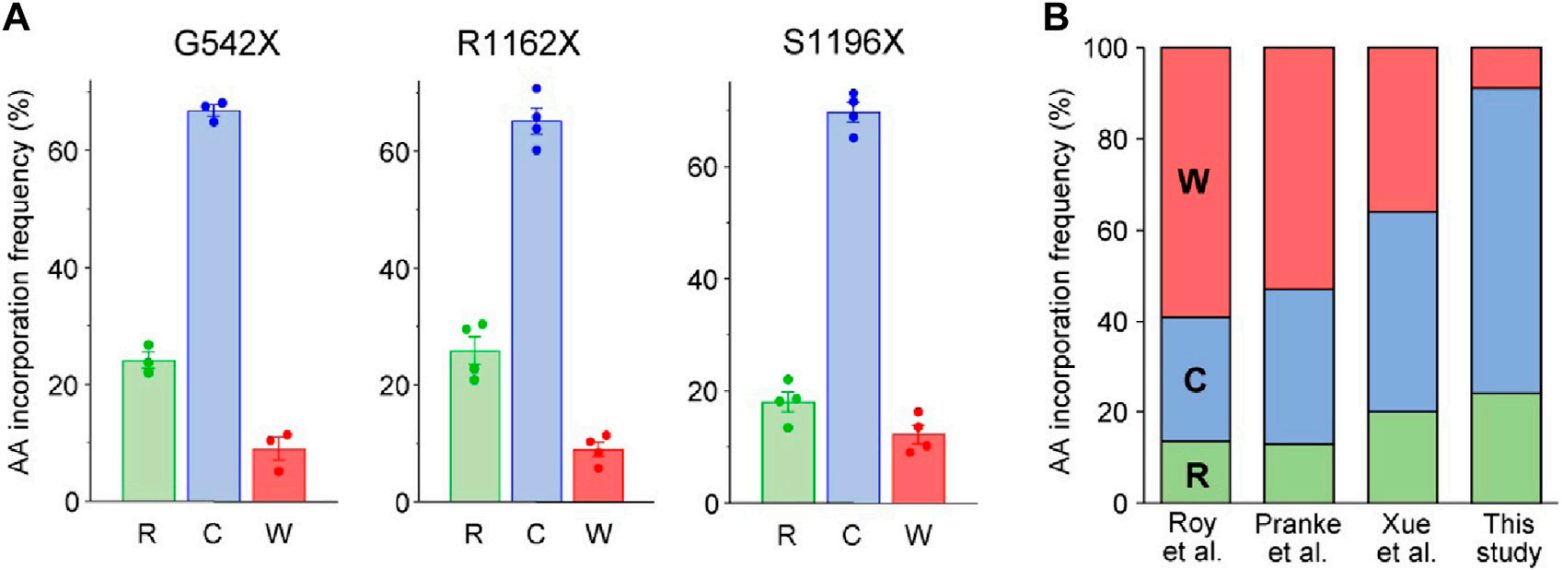
Identification of amino acids incorporated during PTC suppression of CFTR missense variants in the full-length native context using mass spectrometry. **(A)** Quantification of amino acids inserted during G418 suppression (200 μg/mL) and corrector combination treatment (3 µM VX-809, 5 μM C4, 5 µM 4172, 16–18 h at 37°C) of the G542X, R1162X, and S1196X variants expressed in BHK-21 cells. Raw fragmentation spectra of the identified amino acids/peptides are in Supplementary Figure S1. Data are means ± s. e.m. of three to four independent experiments. **(B)** Comparison of percent amino acid incorporations obtained for G542X according to the previous literature Roy et al. (Roy et al., 2016), Pranke et al. (Pranke et al., 2018) and Xue et al. (Xue et al., 2017)) and the current work. The residues inserted in place of the UGA PTC codon are arginine (R—green), cysteine (C—blue), and tryptophan (W—red).

Following the HBH-tag pulldown of CFTR variants from BHK-21 cells, the samples were subjected to tandem MS analysis. MS/MS analysis of the tryptic peptides revealed three possible a. a. insertions in the UGA codon of G542X- and S1196X-CFTR: arginine (R), cysteine (C), and tryptophan (W), (Figure 2A; Supplementary Figure S1). While the a. a.s agree with published literature for UGA codon insertions (Roy et al., 2016; Pranke et al., 2017; Xue et al., 2017), the ratio observed here favors cysteine and disfavors tryptophan incorporations (Figure 2B). These differences in ratios could be attributed to studying PTC suppression in the fully native context, which is more likely to reflect an endogenous scenario, neglecting any bias that may arise due to the use of chimeric constructs of varying lengths. For similar MS/MS analysis on the tryptic peptides of R1162X-CFTR, we observed only two missense populations: R1162C-CFTR and R1162W-CFTR (Supplementary Figure S1). The third possible incorporation at the UGA is arginine. Thus, we hypothesized that there may be rare events wherein the native arginine residue is reincorporated at the 1162nd residue. However, this would evade detection by MS analysis as trypsin cuts at the C-terminal end of arginine and lysine residues, and the resulting peptide R_1158_SVSR_1162_ would be considerably too small to be detected or fragmented by the mass spectrometer. To test this hypothesis, we switched to using chymotrypsin, an enzyme that cleaves predominantly at hydrophobic residues, to avoid a detection bias upon Arg incorporation. Concordantly, we detected and quantified the Arg-incorporated population of R1162, which corresponds to ∼25% of the full-length proteins (Figure 2A). To the best of our knowledge, this is the first time that the reincorporation of the native residue upon PTC suppression is reported.

### 3.3 Consequence of the a. a. misincorporation on the functional expression of G542X and R1162X variants

**FIGURE 3:**
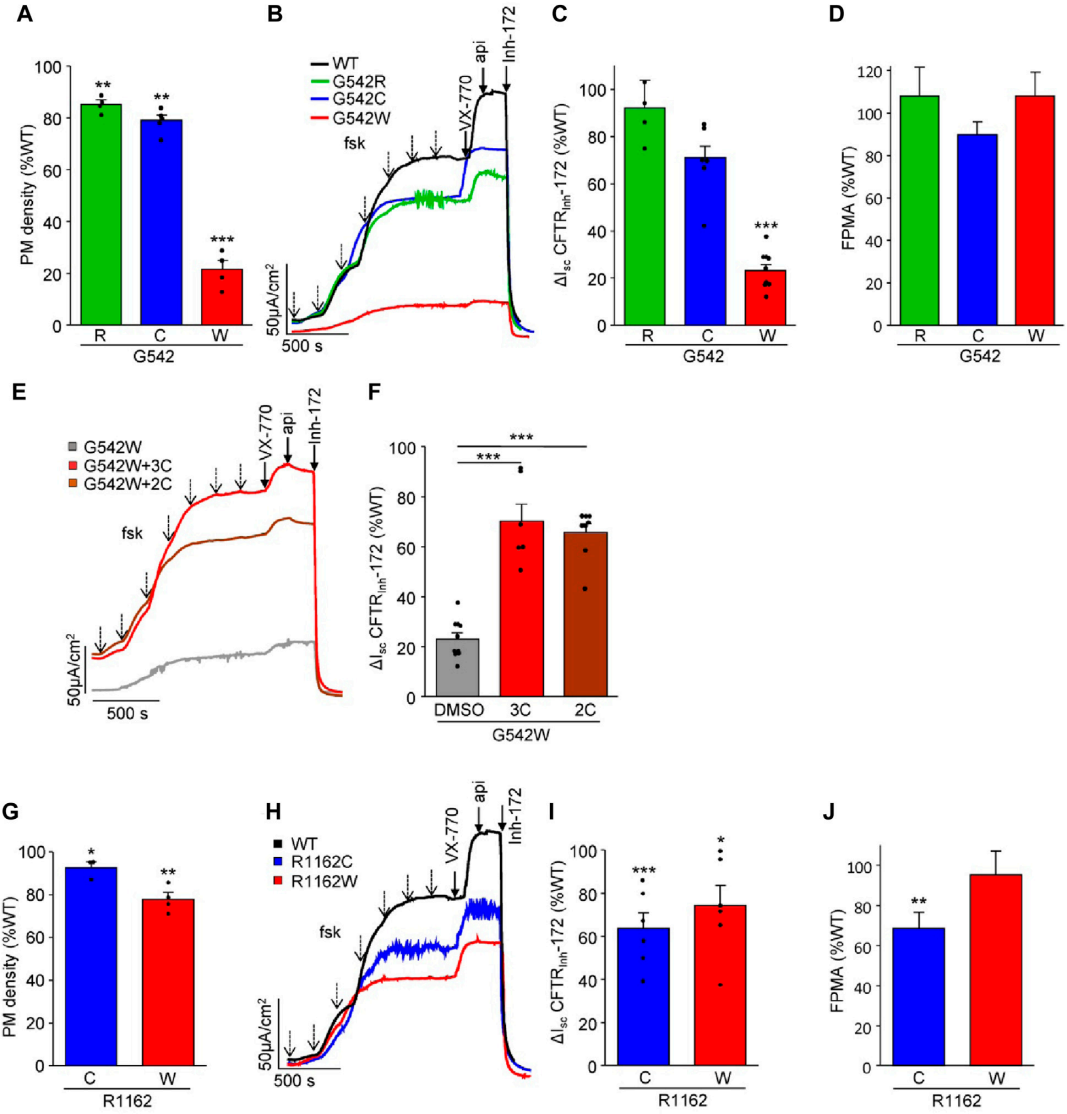
Functional characterization of missense CFTR variants mimicking amino acid misincorporation at positions G542 and R1162 in CFBE41o- cells. **(A)** Cell surface expression of G542 missense variants shown as a percentage of WT-CFTR and normalized to mRNA expression (n = 4–5). **(B,C)** Representative I_sc_ traces **(B)** and quantification [**(C)**, n = 6–10, *CFTR* mRNA-normalized] of CFTR_Inh_-172 inhibited currents of WT-CFTR and G542R, C, and W. I_sc_ were measured in the presence of 100 µM amiloride and a basolateral-to-apical chloride gradient after basolateral permeabilization with amphotericin B (100 µM). CFTR was activated by increasing concentrations of forskolin (dotted arrows—10 nM, 30 nM, 100 nM, 300 nM, 1 μM, 3 μM, 10 µM) and potentiated with 3 µM VX-770 and 50 µM apigenin. **(D)** Fractional PM activity of G542 missense variants. **(E,F)** Representative I_sc_ traces **(E)** and quantification **(F)**, (n = 6–10, *CFTR* mRNA-normalized) of the rescue of G542W pre-treated with triple correctors (3C, 3 µM VX-809 + 10 µM 3151 + 10 µM 4172, 24 h) or double correctors (2C, 3 µM VX-661 + 2 µM VX-445, 24 h). **(G)** Cell surface expression of R1162 missense variants as a percentage of WT-CFTR and normalized to mRNA expression (n = 3–4). **(H,I)** Representative I_sc_ traces **(H)** and quantification **(I)**, n = 6, *CFTR* mRNA-normalized) of the CFTR_Inh_-172 inhibited currents of WT-CFTR and R1162C and W. **(J)** Fractional PM activity of R1162 missense variants. The data in **(A, C, F, G, and I)** are means ± s.e.m. of the indicated number of independent experiments. *, *p* < 0.05; **, *p* < 0.01; ***, *p* < 0.001 by unpaired, two-tailed Student’s t-test.

To investigate the impact of a. a. misincorporations upon PTC suppression on CFTR protein expression and function in G542X and R1162X mutants, we introduced two or three misincorporated a. a. residues at the 1162nd and 542nd positions, respectively. S1196X was not included in these studies as the native-like truncated molecule constituted the largest fraction and was amenable to rescue by correction (Figures 1D, H) (Sharma et al., 2018). The mRNA and protein expression levels of these CFTR variants in CFBE41o- cells were examined by qPCR and PM density measurement, respectively (Figures 3A, G; Supplementary Figure S2). While the mRNA expression was comparable or WT-like across all variants, the mRNA-normalized PM expression of G542W-CFTR was significantly attenuated to ∼20% of the WT, suggesting a folding defect. Similarly, the G542W mutant conferred short-circuit currents (I_sc_) corresponding to only ∼20% of the WT, whereas the G542R and G542C missense variants produced WT-like I_sc_ (Figures 3B, C). The fractional PM activity, defined as I_sc_ divided by PM density, which is a proxy for the channel open probability, was WT-like (Figure 3D), indicating that these mutants do not exhibit evident gating defects. Nevertheless, the functional expression defect of the G542W mutant was significantly rescued by treatment with the corrector combinations 3C (VX-809 + 3151+4172) or 2C (VX-661+VX-445) (Figures 3E, F).

Both the R1162C and R1162W missense substitutions resulted in a mild folding defect, exemplified by the ∼10–20% reduction in PM density (Figure 3G). R1162W showed a proportionally reduced I_sc_. However, the R1162C conferred only ∼60% of WT function (Figures 3H, I), suggesting a mild gating defect that was incompletely reverted by VX-770 (Figures 3H–J).

Collectively, our findings suggest that the nonsense suppression of G542X and R1162X, with the exception of the low abundance G542W variant, results in the expression of missense variants exhibiting only mild folding and functional defects. Thus, NMD suppression and readthrough therapy are likely the determining factors for achieving clinically relevant channel activity.

### 3.4 Readthrough therapy is the limiting factor for the correction of G542X and R1162X-CFTR functional expression defects

**FIGURE 4:**
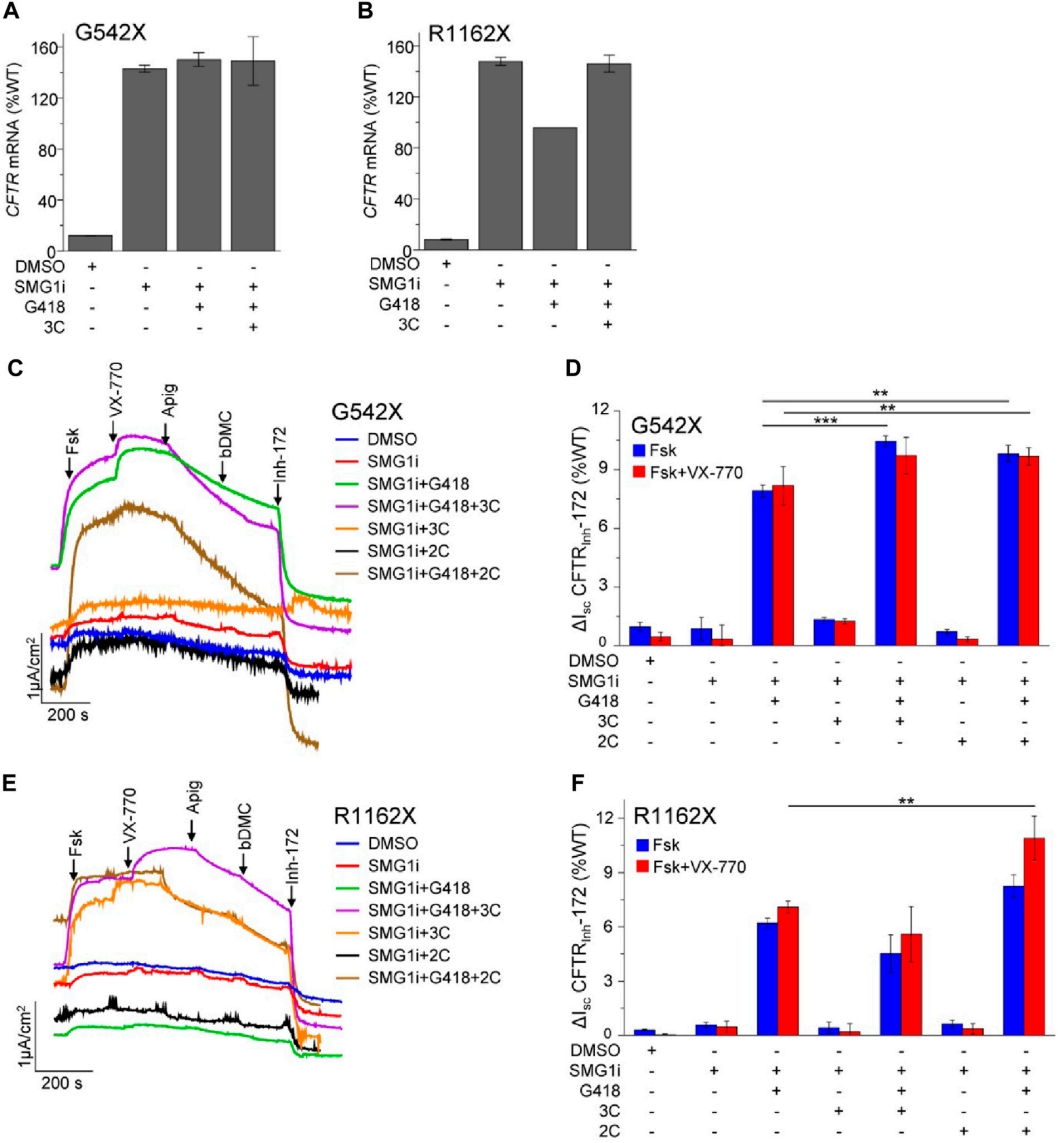
NMD inhibition and readthrough partially rescue the functional expression of G542X and R1162X in 16HBEge cell lines. **(A,B)** *CFTR* mRNA expression of G542X **(A)** and R1162X **(B)** in 16HBEge cell lines determined by qPCR and expressed as the percentage of endogenous WT-*CFTR* mRNA expression in 16HBE14o-. The cells were treated for 24 h with the following compounds alone or in combination: DMSO, 1 µM SMG1i, 200 μg/mL G418, and 3C—3 µM VX-809 + 10 µM 3151 + 10 µM 4172. Data are mean ± s. e.m. of two to three independent experiments. **(C–F)** Representative I_sc_ traces **(C,E)** and quantification **(D,F)** of CFTR_Inh_-172 inhibited currents of G542X and R1162X in 16HBEge cells upon 24-h treatment with the indicated drugs/small molecules: 200 μg/mL G418, correctors (3C—3 μM VX-809, 10 μM 3151, 10 μM 4172 or 2C - 3 μM VX-661 + 2 μM VX-445) and 1 μM SMG1i. I_sc_ was measured in the presence of 100 µM amiloride and a basolateral-to-apical chloride gradient in intact monolayers. CFTR was activated with forskolin (20 µM) and potentiated with 3 µM VX-770, 50 µM apigenin, and 10 µM bDMC. Data are means ± s.e.m. of three to four independent experiments. **, *p* ≤ 0.01; ***, *p* ≤ 0.001 by one-way ANOVA followed by Turkey’s *post hoc* test.

To study the CFTR PTC mutants within their native genetic context, we used the bronchial epithelial cell lines 16HBE14o- that were modified by CRISPR/Cas9 gene editing (16HBEge) to contain the G542X and R1162X mutations (Valley et al., 2019). In these cells, the steady-state *G542X* and *R1162X-CFTR* mRNA levels were 12% and 8% of the WT, respectively (Figures 4A, B). Similar to observations for W1282X (Laselva et al., 2020; Venturini et al., 2021), inhibition of SMG1 restored the G542X and R1162X mRNA expression to or beyond that of the WT-CFTR (Figures 4A, B). The mRNA expression was not substantially influenced by co-treatment with G418 and the 3C corrector combination.

Subsequently, we monitored the CFTR functional rescue upon the combination of NMD inhibition, readthrough, and combinatorial modulator therapy by measuring the I_sc_ in the gene-edited 16HBEge human bronchial epithelial cells. Neither NMD inhibition with the SMG1 inhibitor (SMG1i) alone nor the combination of SMG1i with CFTR correctors was sufficient to achieve a significant current for G542X-CFTR and R1162X-CFTR (Figures 4C–F). However, the combination of SMG1 inhibition with G418 treatment resulted in a significant increase in the G542X-CFTR and R1162X-CFTR current, corresponding to ∼8% and ∼6% of the WT I_sc_, respectively (Figures 4C–F). Co-treatment with the VX-661+VX-445 (2C) corrector combination led to a small but significant increase in the I_sc_, which reached ∼10% and ∼8% of the WT for G542X-CFTR and R1162X-CFTR, respectively. We also investigated whether acute potentiation with VX-770 alone or in combination with the co-potentiators apigenin and bDMC (Veit et al., 2020) would further increase the G542X or R1162X I_sc_; however, this was not observed. Counterintuitively, while the addition of apigenin and bDMC synergistically improved the function of W1282X (Haggie et al., 2017; Phuan et al., 2019), it resulted in reduced I_sc_ for G542X or R1162X mutants. This observation suggests that co-potentiation may be detrimental or not required for these specific mutants.

These results jointly suggest that both NMD suppression and promotion of readthrough are requirements for G542X and R1162X therapy. While SMG1i treatment restored the mRNA of both mutants to the WT level, the maximally achieved currents were <10% of the WT. This cannot be explained by a folding and/or gating defect resulting from a. a. misincorporation since the most frequent variants only exhibited mild folding/gating defects, and treatment with combinations of correctors and potentiators resulted only in a small current increase. Therefore, low efficacy readthrough suppression and/or impeded translational initiation are likely the limiting factors to restore functional correction of G542X and R1162X-CFTR.

## 4 Discussion

The residual functionality and the responsiveness to various CFTR modulators, NMD suppressors, and readthrough drugs are determined by the susceptibility of a PTC mutant to NMD, the stimulated SCR of near-cognate tRNAs, as well as the folding and/or gating defect of resulting missense variants. This level of complexity makes *a priori* estimation of a PTC mutant responsiveness to pharmacological interventions challenging, suggesting that precision medicine approaches are necessary to empirically define the most effective drug combination. Here, we exemplify such a mutation-specific process for two PTC mutants, G542X and R1162X, by determining the steady-state mRNA level, the responsiveness to NMD suppression and studying the level of readthrough product, as well as deciphering the missense a. a. incorporation ratios in the full-length CFTR variants. These measurements were combined with monitoring the folding/gating defects of the missense readthrough CFTR variants and applying these results to determine the most effective drug combination for their functional rescue.

The introduction of the C-terminal HBH-tag (Okiyoneda et al., 2018; Schnur et al., 2019) enabled the selective isolation of the readthrough product, allowing for the estimation of the fraction of full-length *versus* truncated molecules at the cell surface. While the truncated S1196X-CFTR and W1282X-CFTR could be localized to the PM, as reported before (Sharma et al., 2004; Du and Lukacs, 2009; Haggie et al., 2017) the larger truncations G542X and R1162X required readthrough, which could be partially induced by treatment with the aminoglycoside antibiotic G418. The PTC mutants were chosen due to their UGA stop codon, which is more “leaky” and has a higher readthrough efficiency compared to other stop codons (Manuvakhova et al., 2000; Dabrowski et al., 2018; Pranke et al., 2023). The identity and abundance of the (mis)incorporated a. a.s were determined by MS/MS analysis of the affinity-purified full-length channel.

The a. a. incorporation for the UGA codon in presence of aminoglycosides has been previously studied, using minigenes in yeast or HEK-293 expression systems resulting in the relative incorporation of tryptophan (36%–59%), cysteine (27%–44%) and arginine (13%–20%) (Roy et al., 2016; Xue et al., 2017; Pranke et al., 2018). However, we obtained different G418-induced a. a. incorporation ratios into three UGA stop codons in the G542X-, R1162X- and S1196X-CFTR: ∼65–70% of the isolated molecules contained cysteine at the PTC site, ∼20–25% contained arginine, and only ∼10% contained tryptophan. This discrepancy could reflect differences in tRNA abundances between yeast or HEK-293 cells and the BHK-21 cells used in this study, which have tRNA levels similar to human CFBE41o- cells (Figure 1A). Post-transcriptional modification of tRNAs, which is in part species and cell-type specific (Pinkard et al., 2020), can modulate translational misreading errors (Saleh and Farabaugh, 2023). Thus, the choice of cell model beyond tRNA abundance may influence the a. a. incorporation at PTC sites. The a. a. incorporation at PTC sites is also influenced by both near and distant mRNA sequence contexts (Xue et al., 2017; Wangen and Green, 2020). While nucleotides at the +1 and +4 positions are known to affect interactions between the mRNA and translational machinery (McCaughan et al., 1995; Jungreis et al., 2011; Loughran et al., 2014; Beznoskova et al., 2016), relatively distant nucleotides in positions +5, +6, +8, +9 (Dabrowski et al., 2015) and −1, −2–3 positions upstream of the PTC (Tork et al., 2004; Dabrowski et al., 2018) could also influence readthrough. In line, cysteine, tryptophan, and leucine (instead of arginine) were inserted during the G418-mediated PTC suppression of the W1282X-CFTR UGA codon in the context of its three upstream and downstream CFTR codons (Xue et al., 2017). The unexpected effect of the local sequence context on a. a. incorporation has recently been shown for the UGA stop codon of G550X-CFTR, for which tryptophan was identified as the sole missense substitution in a reporter protein (Chen et al., 2023). Intriguingly, this UGA has identical nucleotides at −1 and +1 positions. Beyond the local sequence context, posttranslational modifications of the Rps23 ribosomal protein in the 40S subunit (Loenarz et al., 2014), as well as certain mRNA and peptide features, were also identified to influence translation termination and readthrough efficiency (Mangkalaphiban et al., 2021).

The difference in activity of the G542X missense variants exemplifies the importance of correctly identifying the relative ratios of incorporated a. a.s. While the low abundance G542W variant exhibits a severe folding defect and highly depends on CFTR corrector treatment, the more abundant G542C and G542R variants have WT-like activity. Consistently, CFTR corrector treatment only modestly increased the functional rescue of the G418-induced readthrough product of G542X in 16HBEge epithelia.

An additional factor in the rescuability of CFTR with PTCs is the susceptibility of their mRNA to NMD. Consistent with published results (Venturini et al., 2021), the *G542X-CFTR* and *R1162X-CFTR* mRNA abundance, reduced to <15% of the WT, could be largely normalized by NMD suppression with an SMG1 inhibitor drug. For functional rescue, however, readthrough induction with G418 was indispensable. Nevertheless, the resulting currents, even in the presence of corrector and potentiator combinations, did not exceed ∼10% of the WT. This phenomenon could conceivably be explained by a combination of processes, including the low efficacy of SCR, the reduced translational initiation due to non-optimal codon usage at the stop codon (Barrington et al., 2023), and the co-activation of protein quality control mechanisms by the NMD (Chu et al., 2021; Udy and Bradley, 2022).

It is important to note that the high toxicity prevents the of aminoglycosides usage for CF therapy (Bedwell et al., 1997; Nagel-Wolfrum et al., 2016; Dabrowski et al., 2018). To overcome this challenge, chemically modified second-generation aminoglycosides with reduced toxicity have been synthesized (Sabbavarapu et al., 2016). One of these compounds, ELX-02, showed promise in preclinical models (Kerem, 2020; Crawford et al., 2021; Pranke et al., 2023). In clinical trials the safety of ELX-02 was established (Leubitz et al., 2021), but it achieved clinically relevant improvement in lung function in only a subset of patients with class I mutations (https://investors.eloxxpharma.com/news-releases/news-release-details/eloxx-pharmaceuticals-announces-final-data-assessment-phase-2). This may reflect variability in mRNA levels between patients carrying the same PTC mutation (Linde et al., 2007b; Clarke et al., 2019), which lead to patient specific responses to readthrough drugs and suggest patient-specific variability in NMD. However, a nonselective approach to NMD inhibition may not be a viable therapeutic strategy since the NMD pathway not only degrades PTC-containing mRNAs but also serves as a post-transcriptional regulatory mechanism for 10%–20% of the transcriptome (Yi et al., 2021; Sanderlin et al., 2022). Investigations of NMD branch-specificity of PTC-containing CFTR transcripts, however, may offer more targeted interventions (Sanderlin et al., 2022).

In conclusion, by comprehensively analyzing PTC transcript abundance and amino acid missense incorporations into PTC translation products, we established a framework for a deeper understanding of molecular complexities associated with PTC mutants in CF. This approach could be extended to a broader spectrum of CFTR mutations, allowing for the development of more tailored and comprehensive therapeutic strategies. Beyond CF, the identified principles offer potential applications in a diverse range of diseases linked to nonsense mutations.

## Data Availability

The raw MS/MS data presented in the study are deposited in the Zenodo repository (https://zenodo.org/records/10928064 or doi: https://doi.org/10.5281/zenodo.10928064). All other original contributions presented in the study are included in the article/Supplementary Material, further inquiries can be directed to the corresponding author.
