# Supplementary material for: Readthrough-induced misincorporated amino acid ratios guide mutant-specific therapeutic approaches for two CFTR nonsense mutations
Source: Front Pharmacol. 2024 Apr 25;15:1389586. doi: 10.3389/fphar.2024.1389586 (PMC11079177; doi:10.3389/fphar.2024.1389586)
Supplement: Supplementary file 1 [file DataSheet1.PDF]

Supplementary Information

for

Readthrough-induced misincorporated amino acid ratios guide mutant-specific therapeutic approaches for two CFTR nonsense mutations

Aiswarya Premchandar, Ruiji Ming, Abed Baiad, Dillon F. Da Fonte, Haijin Xu, Denis Faubert, Guido Veit, and Gergely L. Lukacs

This file contains:

Supplementary Figure 1–2.

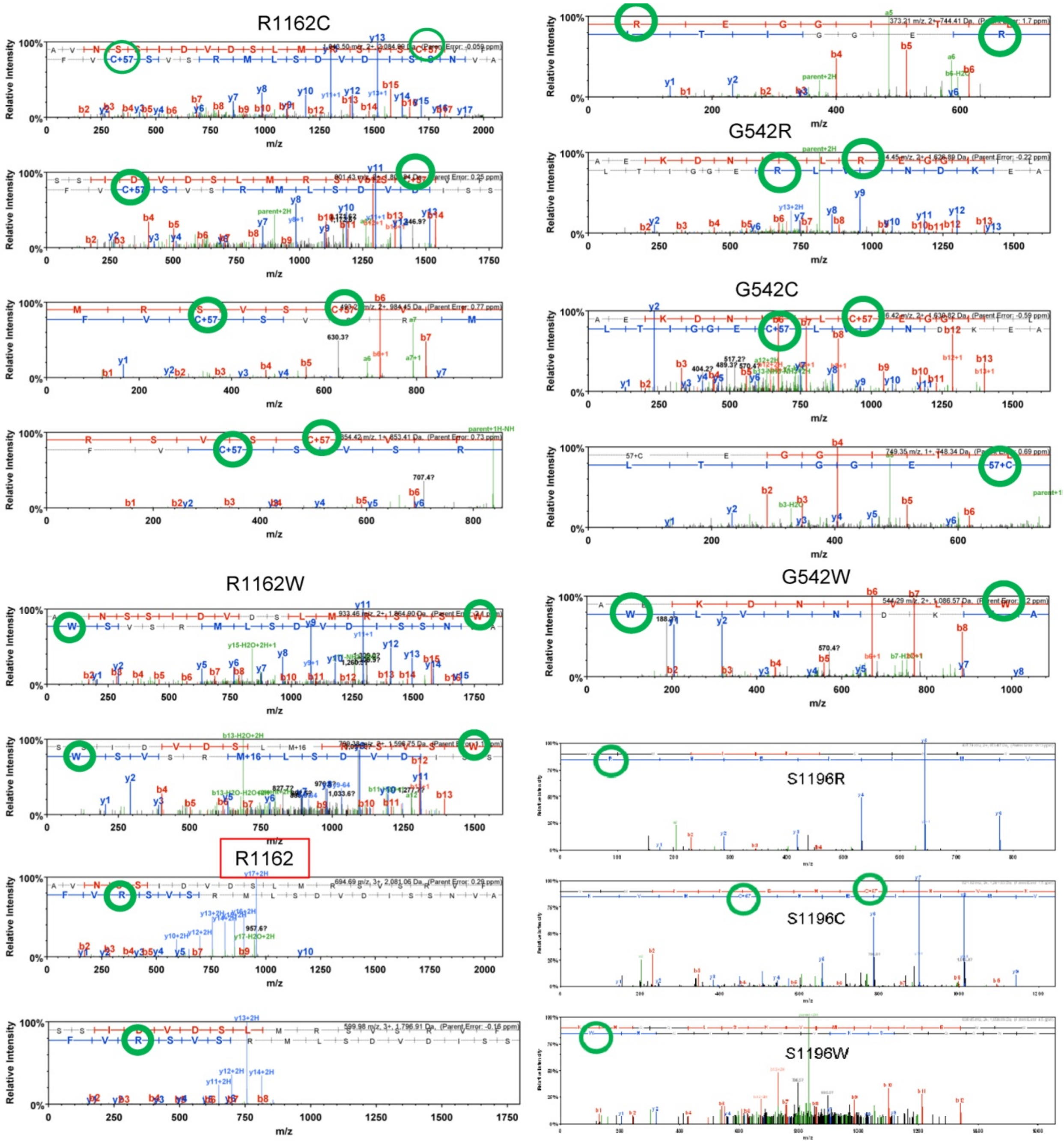

Supplementary Figure 1. Representative MS/MS fragmentation spectra. Representative list of MS/MS fragmentation spectra obtained from Scaffold™ 5.0 for each identified a.a. misincorporation (circled in green). The b- (blue) or y-ions (red) are based on charge retention at the N- or C-terminal fragments, respectively. Fragmentation evidence from b- and/or y- ions reflects upon the good spectral quality and higher confidence of a.a. identification.

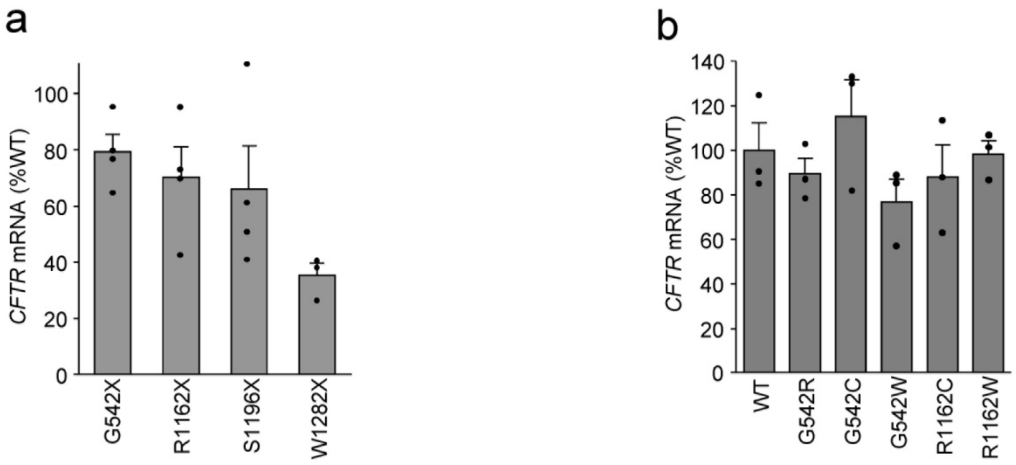

Supplementary Figure 2. Steady-state *CFTR* mRNA expression. (a) Steady-state *CFTR* mRNA expression in BHK-21 for G542X, R1162X, S1196X, and W1282X mutants as determined by qPCR, expressed as the percentage of WT-*CFTR* mRNA levels (n = 3–4). **(b)** Steady-state *CFTR* mRNA expression in CFBE41o- for G542X and R1162X missense mutations as determined by qPCR, expressed as the percentage of WT-*CFTR* mRNA levels (n = 4). Data are means ± s.e.m. of the indicated number of independent experiments.
